# Association between Serum Vitamin B12 and Global DNA Methylation in Colorectal Cancer Patients

**DOI:** 10.3390/nu12113567

**Published:** 2020-11-20

**Authors:** Hatim Boughanem, Pablo Hernandez-Alonso, Alberto Tinahones, Nancy Babio, Jordi Salas-Salvadó, Francisco J. Tinahones, Manuel Macias-Gonzalez

**Affiliations:** 1 Department of Endocrinology and Nutrition, Institute of Biomedical Research in Malaga (IBIMA), Virgen de la Victoria University Hospital, 29010 Malaga, Spain; h.b.boughanem@gmail.com (H.B.); pablo1280@gmail.com (P.H.-A.); albertotruano@gmail.com (A.T.); 2 Unitat de Nutrició Humana, Departament de Bioquímica i Biotecnología, Universitat Rovira i Virgili, 43201 Reus, Spain; nancy.babio@urv.cat (N.B.); jordi.salas@urv.cat (J.S.-S.); 3 Institut d’Investigació Sanitària Pere Virgili (IISPV), Hospital Universitari San Joan de Reus, 43204 Reus, Spain; 4 CIBER in Physiopathology of Obesity and Nutrition (CIBEROBN), Instituto de Salud Carlos III, 28029 Madrid, Spain

**Keywords:** vitamin B12, DNA methylation, *LINE1*, colorectal cancer

## Abstract

Vitamin B12 has been widely related to methionine metabolism, which is an essential component for biological methylation reactions, including DNA methylation. However, the relationship between vitamin B12 and DNA methylation is still controversial. In addition, there is increasing evidence for the association between vitamin B12 and the risk of colorectal cancer (CRC), although results of this association need to be assessed with caution. For this purpose, we hypothesized that serum vitamin B12 could be associated with global DNA methylation in the CRC context. To test this hypothesis, we studied the association between global DNA methylation through long interspersed nuclear element-1 (*LINE1*) in CRC patients under the 25th percentile of serum vitamin B12. We found that the high vitamin B12 group had low *LINE1* methylation in both tumor area and peripheral blood mononuclear cells (PBMCs) than the low serum vitamin B12 group. *LINE1* methylation levels were significantly lower in tumor area compared to the adjacent tumor-free area, only in the high vitamin B12 group. *LINE1* methylation in visceral adipose tissue (VAT) and PBMCs were correlated with tumoral, inflammatory, and insulin metabolism markers. However, the interaction between *LINE1* methylation and vitamin B12 levels was associated with neoadjuvant therapy in the regression analysis only in men, suggesting a beneficial relationship. In conclusion, our results reported an inverse association between DNA methylation and vitamin B12 in the CRC context, which suggests that vitamin B12 may be implicated in an epigenetic state or mediation in CRC.

## 1. Introduction

Vitamin B12 plays essential roles in the metabolism of DNA methylation, through its participation in the homocysteine metabolism. It is required by the methionine synthase, a vitamin B12-dependent enzyme that catalyzes the formation of methionine from homocysteine using 5-methyltetrahydrofolate, which is subsequently converted to tetrahydrofolate [1]. Methionine, in turn, is converted to 5-adenosylmethionine, being an essential component for biological methylation reactions, including DNA and histone methylation [2]. However, the relationship between vitamin B12 and DNA methylation remains unclear.

Previous studies have reported associations between vitamin B12 and DNA methylation [3]. An epigenome-wide study reported that vitamin B12 supplements influence the regulation of several genes, in fact, 589 differentially methylated CpGs (Cytosine-phosphate-guanine) (DMPs) and 2892 differentially methylated regions (DMRs) were reported [4]. In addition, long-term supplementation with folate and vitamin B12 resulted in changes of 162 DMPs, although comparisons of the DNA methylation changes in the participants receiving supplements of folic acid and vitamin B12 versus placebo reported one single differentially methylated position [5]. Even a large-scale genome-wide DNA methylation study did not find correlation between vitamin B12 intake and DMPs [6]. Furthermore, the relationship between both dietary intake and supplementation with vitamin B12 and DNA methylation is still controversial. Importantly, there is a lack of studies evaluating the link between serum levels of vitamin B12 and DNA methylation.

On the other hand, although vitamin B12 deficiency has been widely associated to colorectal cancer (CRC), the mechanisms underlying this relationship have not been identified yet [7]. A nested-case referent prospective study showed that plasma vitamin B12 concentration was inversely associated with the risk of rectal cancer, although its association with the risk of colon cancer was less apparent [8]. These results have been confirmed by another prospective case-control study, in which circulating vitamin B12 levels were inversely associated with rectal cancer risk but not with overall colorectal cancer [9]. However, in a multiethnic cohort study, no significant associations were reported between plasma vitamin B12 levels and CRC risk [10], whereas vitamin B12 and folate supplementation were related with an increased risk of CRC [11].

Therefore, the role of vitamin B12 on DNA methylation and CRC development is largely unknown but promising, since there is evidence supporting this specific link between B12 and CRC through epigenetic conditions. For example, it has been shown that serum vitamin B12 was associated with the methylation of the promoter of genes related with colorectal carcinogenesis, such as mutL homolog 1 (MLH1) and O-6-methylguanine-DNA methyltransferase (MGMT) in elderly subjects [12]. In the case that these associations exist, it would be interesting to test the potential preventive effect of B12 administration on CRC risk.

In the present study, we hypothesized that circulating levels of vitamin B12 may be associated with global DNA methylation in the CRC context and this association may be related with clinical cancer outcomes. For this purpose, we studied the association between methylation of long interspersed element-1 (*LINE1*) as a marker of global DNA methylation and serum vitamin B12 in different biological samples, such as peripheral blood mononuclear cells (PBMCs)—as a non-invasive source tissue, visceral adipose tissue (VAT)—as an active metabolic tissue, as well as tumoral tissues. We also investigated the relationship between *LINE1* methylation and several clinical and biochemical outcomes to evaluate the additional relationship between biochemical and anthropometric variables, DNA methylation, and serum vitamin B12 in CRC patients.

## 2. Materials and Methods

### 2.1. Study Design and Participants

Our study included 80 CRC patients recruited at the “Virgen de la Victoria” University Hospital of Malaga (Málaga, Spain), from 2011 to 2014. CRC patients were categorized into two groups based on the 25th percentile in serum vitamin B12 cut-off. Nineteen CRC patients were below the 25th percentile (<25th percentile), and 61 were above the 25th percentile of vitamin B12 (≥25th percentile). The normal range for vitamin B12 is generally between 200 and 900 pg/mL. A range between 200 to 300 pg/mL is considered borderline and below 200 pg/mL is considered a low value [13]. In our cohort, the cut-off value of the 25th percentile of vitamin B12 was 261 pg/mL, which was located within the borderline values, and the mean of the low vitamin B12 group was 199.79 (Standard deviation (SD): 49.14) pg/mL, which was also located under 200 pg/mL and considered a low value. CRC patients were diagnosed by oncologists by means of a colonoscopy and biopsy. Pathologists classified tumor characteristics based on histological features. All diagnosed CRC patients underwent surgery with curative intention, by hemicolectomy, lower anterior resection with ileostomy (caused by a CRC carcinoma), followed by a total mesocolorectal excision. Adjacent tumor-free area was also collected and compared with the tumor area. Neoadjuvant treatment was conducted according to the local protocols. CRC patients received neoadjuvant treatment consisting of chemoradiation treatments with pelvic radiotherapy 50 Gray (Gy) (2 Gy/fraction) and concomitant administration of fluoropyrimidine-based chemotherapy, followed by total mesorectal excision after 6–8 weeks. Biopsy and tumor samples were fixed by using formalin-fixed paraffin-embedded (FFPE). Epiploic VAT from CRC patients was obtained during surgery, and washed using a saline solution, and frozen in liquid nitrogen. The VAT was stored at −80 °C until DNA extraction. PBMCs were obtained by blood (whole blood, plasma dipotassium ethylenediaminetetraacetic acid (EDTAK2) primary tube BD Vacutainer^®^ EDTA plasma Tube) (Becton, Dickinson and Company, Franklin Lakes, NJ, USA)) centrifugation for 15 min at 1800× *g* and frozen at −80 °C until DNA extraction.

The exclusion criteria for the present study were patients with history of familial adenomatous polyposis, history of nonpolyposis colorectal cancer, cardiovascular disease events or inflammatory diseases, presence of high insulin resistance (homeostatic model assessment for insulin resistance (HOMA-IR) > 5) [14,15], type 2 diabetes (T2D), and systemic or local infectious diseases. Patients receiving antidiabetic or hypocholesterolemia treatments were also excluded. We also excluded patients consuming more than 20 g/day of ethanol. The study was approved by the Ethical Committee of Virgen de la Victoria University Hospital (Málaga, Spain) (registration number 0311/PI7) and written informed consent was obtained from all CRC patients.

### 2.2. Biochemical Determinations

Fasting serum samples were obtained after centrifugation for 15 min at 1800× *g*. Serum total cholesterol, high-density lipoprotein cholesterol (HDL-c), triglycerides, and glucose concentrations were determined by means of a Dimension Autoanalyzer (Dade Behring Inc., Deerfield, IL, USA). Low-density lipoprotein cholesterol (LDL-c) was calculated using the Friedewald equation [16]. Insulin levels were measured by a radioimmunoassay method using BioSource International Inc. (Camarillo, CA, USA). HOMA-IR was estimated using the following equation: HOMA-IR = fasting insulin (μIU/mL) × fasting glucose (mM)/22.5 [17]. Serum vitamin B12 was quantified by chemiluminescent immunoassay (Roche Diagnostics GmbH, Penzberg, Germany). C-reactive protein (CRP) was measured in a Dimension autoanalyzer (Dade Behring Inc., Deerfield, IL, USA). Insulin growth factor type 1 (IGF-1) was determined using a Human IGF1 ELISA Kit (Abcam, Madrid, Spain). Carcinoembryonic antigen (CEA) and carbohydrate antigen 19.9 (CA 19.9) were measured by ELISA (DRG diagnostics, Marburg, Germany).

### 2.3. DNA Extraction from Biological Samples, Bisulfite Reaction, and Pyrosequencing

DNA was isolated from PBMCs using a Qiamp DNA blood mini kit (Qiagen GmbH, Hilden, Germany), from VAT using a Qiamp DNA Tissue Kit (Qiagen GmbH, Hilden, Germany), and from 10 sections of 14 μm of paraffin samples from the tumor area and adjacent-tumor free area, using a Qiamp DNA FFPE Tissue Kit under the instructions of the manufacturer (Qiagen GmbH, Hilden, Germany), with a xylene wash to remove the paraffin. DNA integrity was checked by a nanodrop at A 260/A 280 and A 260/A 230 and stained agarose gels. We used 2 μg of isolated DNA for bisulfite reaction. This reaction was performed using an EpiTect Fast Bisulfite Kit (Qiagen GmbH, Hilden, Germany), according to the instructions of the manufacturer. We measured the promoter methylation of *LINE1*. The primer sequences and additional information about CpG sites are detailed in Supplementary Table S1, and the reverse primer was biotinylated, in order to purify the final PCR product, using Sepharose beads (Sigma Aldrich, Madrid, Spain). PCR for bisulfited DNA was made using 0.2 nM of each primer and the PCR pyromark kit (Qiagen GmbH, Hilden, Germany). The final volume was 25 μL. Finally, 20 μL of the PCR products were pyrosequenced using the PyroMarkTMQ96 ID Pyrosequencing System (Qiagen GmbH, Hilden, Germany), using 0.4 μM of sequencing primer. The methylation average was expressed as the percentage of methylated cytosine over the sum of methylated and unmethylated cytosines for six CpGs at the *LINE1* sequence. Inter-assay precision (coefficient of variation (CV)) was 2.5% and intraassay (CV) for the CpG methylation and unmethylated cytosines was 1.0%. Non-CpG cytosine residues were used as built-in controls to verify bisulfite conversion. We also included unmethylated and methylated DNA as controls in each run (New England Biolabs, Ipswich, MA, USA).

### 2.4. Statistical Analysis

The continuous variables were presented as mean ± standard deviation (SD). Categorical variables were shown as number (percentage). We categorized our subjects’ vitamin B12 circulating levels as low (<25th percentile of vitamin B12) or high (≥25th percentile of vitamin B12). Welch’s two sample test was used to determine significant differences in continuous variables due to their non-normal distribution and unequal sample size of the two vitamin B12 groups. A Pearson’s correlation analysis was performed to evaluate the association between variables according to vitamin B12 groups. A Bonferroni correction was applied per group, methylation location, and variables’ co-correlation (i.e., age, body mass index (BMI), glucose/insulin metabolism, and lipid profile; or the set of tumoral and inflammatory markers). A paired *t*-test was performed to compare means between the tumor and adjacent tumor-free area. Linear and logistic regression analyses were performed to determine whether the variability of variables is explained by other variables, under the 25th percentile of vitamin B12. Data analyses were performed using the R v3.5.1 software (Integrated Development for R. RStudio, PBC, Boston, MA, USA), and significance was set at *p* < 0.05 unless otherwise stated.

## 3. Results

### 3.1. General Characteristics of CRC Patients by Serum Vitamin B12 Status

The baseline general, anthropometric, and biochemical characteristics of CRC patients are summarized in Table 1 and Supplementary Table S2. There were no statistically significant differences in age, anthropometric variables, glucose metabolism parameters, most of the lipid profile parameters, and tumoral and inflammation markers when we compared participants with high serum vitamin B12 levels (HVB_12_) and those with low levels (LVB_12_). However, LDL-c and IGF1 levels were significantly higher in the HVB_12_ group than in the LVB_12_ group (*p* = 0.043 and *p* = 0.008, respectively). We also observed significant differences in sex towards a more equal sex distribution in the HVB_12_ group compared to the LVB_12_ group. As expected, the mean value of serum vitamin B12 in the LVB_12_ group was significantly lower (199.79 ± 49.14 pg/mL) compared to those in the HVB_12_ group (507.02 ± 337.83 pg/mL).

### 3.2. Association between Global Methylation Study (LINE1) and Circulating Levels of Vitamin B12 in CRC Patients

**Figure 1:**
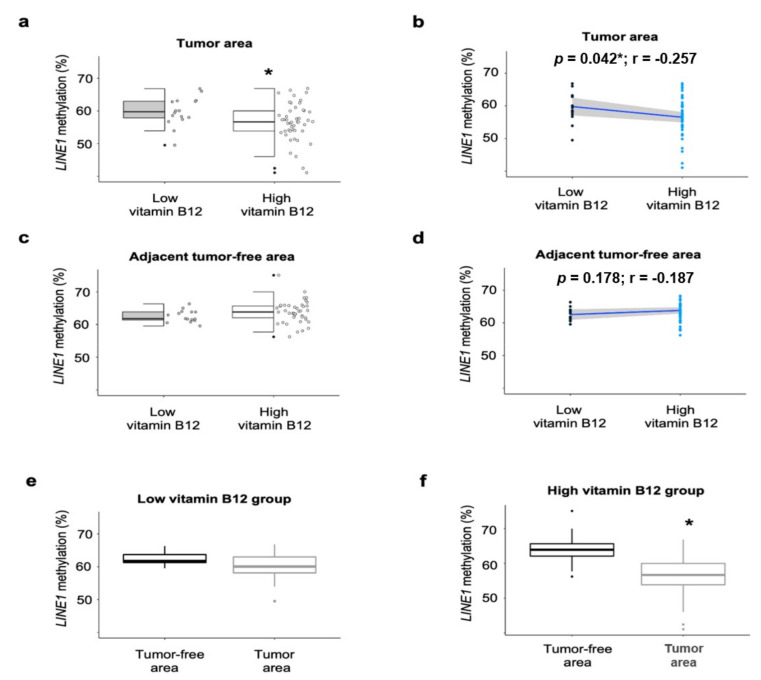
*LINE1* methylation in CRC tumor and the 25th percentile of vitamin B12 in CRC patients. Methylation analyses of *LINE1* in (**a**,**b**) CRC tumor area, (**c**,**d**) adjacent tumor-free area, and (**e**,**f**) comparison between tumor and tumor-free area, according to the 25th percentile of vitamin B12 (low serum vitamin B12 = 19; high serum vitamin B12 = 61). Methylation percentages are presented as median (interquartile range) (in **a**,**c**,**e**,**f**) or individual as individual dots (in **b**,**d**). Significant differences between the two groups were assessed using the Welch’s two sample test for the (**a**,**c**) (* *p* < 0.05). Paired *t*-test was performed to compare means between tumor and adjacent tumor-free area for the (**e**,**f**) (* *p* < 0.05). Pearson correlation was used to determine association between *LINE1* methylation and serum vitamin B12 in the (**b**,**d**). Abbreviations: *LINE1*: Long interspersed nuclear element-1; r: Correlation coefficient; CRC: Colorectal cancer.

We evaluated the association between circulating vitamin B12 and *LINE1* methylation in CRC patients, according to circulating levels of vitamin B12, in CRC tumor and adjacent tumor-free areas, PBMCs, as well as VAT. *LINE1* methylation in the tumor area was lower in the HVB_12_ group compared to the LVB_12_ group (*p* = 0.023) (Figure 1a). However, *LINE1* methylation in the adjacent tumor-free area did not significantly differ between the HVB_12_ and LVB_12_ groups (Figure 1c). *LINE1* methylation in the LVB_12_ group neither differed between the tumor and adjacent tumor-free area (Figure 1e). Nevertheless, *LINE1* methylation in the tumor area was significantly decreased compared to the tumor-free area in only the HVB_12_ group (*p* < 0.001) (Figure 1f).

**Figure 2:**
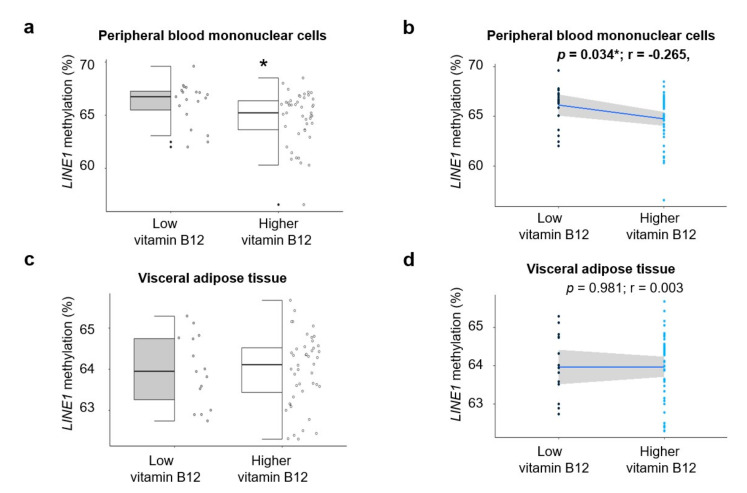
*LINE1* methylation in PBMCs and VAT and the 25th percentile of vitamin B12 in CRC patients. Methylation analyses of *LINE1* in (**a**,**b**) peripheral blood mononuclear cells, (**c**,**d**) visceral adipose tissue, according to the 25th percentile of vitamin B12 (low serum vitamin B12 = 19; high serum vitamin B12 = 61). Methylation percentages are presented as median (IQR) (in **a**,**c**) or individual as individual dots (in **b**,**d**). Significant differences between the two groups were assessed using the Welch’s two sample test for (**a**,**c**) (* *p* < 0.05). Pearson correlation was used to determine the association between *LINE1* methylation and serum vitamin B12 in (**b**,**d**) (* *p* < 0.05). Abbreviations: *LINE1*: Long interspersed nuclear element-1; PBMCs: peripheral blood mononuclear cells; VAT: Visceral adipose tissue; r: Correlation coefficient; CRC: Colorectal cancer.

Similar results were found in PBMCs. Our results showed that the HVB_12_ group had significantly lower methylation levels of *LINE1*, when compared to the LVB_12_ group (*p* = 0.046) (Figure 2a). However, *LINE1* methylation levels in VAT were not statistically significant between the HVB_12_ and LVB_12_ groups (Figure 2c).

### 3.3. Association between Methylation of LINE1 and Biochemical Variables According to the 25th Percentile of Vitamin B12

To study the relationship between *LINE1* methylation in the tumor area, tumor-free area, PBMCs, and VAT and biochemical variables, we performed a Pearson correlation analysis. As shown in Table 2, in the LVB_12_ group, *LINE1* methylation in PBMCs was negatively correlated with HOMA-IR (r = −0.53; *p* = 0.034). In the HVB_12_ group, *LINE1* methylation in VAT was negatively associated with both insulin (r = −0.32; *p* = 0.032) and HOMA-IR (r = −0.31; *p* = 0.040).

### 3.4. Association between Methylation of LINE1 and Tumoral and Inflammatory Markers According to the Circulating Levels of Vitamin B12

**Figure 3:**
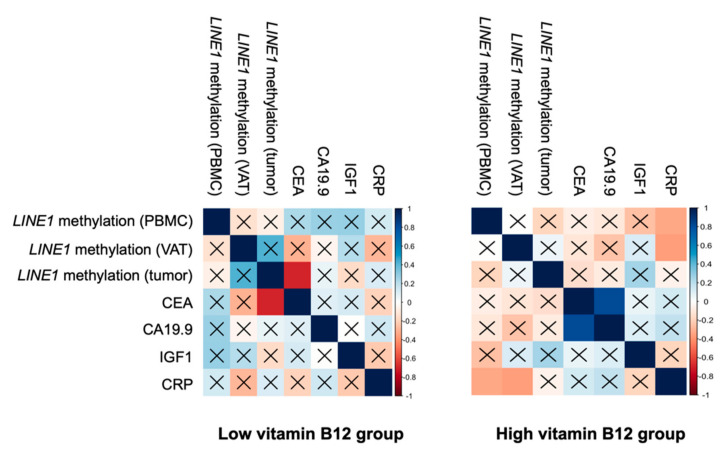
Association between methylation of *LINE1* and tumoral and inflammatory markers according to serum vitamin B12**.** Association between *LINE1* methylation and tumoral and inflammatory markers according to serum vitamin B12 (according to the 25th percentile). The colors are assigned according to the correlation coefficient. Pearson’s correlation was performed to determine the correlation between methylation analyses. Data are expressed as a correlation coefficient. Colored squares without ×, indicate statistically significant between methylated genes and variables according to the Pearson’s correlation test (*p* < 0.05) and after Bonferroni correction (*p* < 0.025). Abbreviations: *LINE1*, Long interspersed nuclear element-1; CEA, carcinoembryonic antigen; CA 19.9, carbohydrate antigen 19.9; IGF1, insulin growth factor type 1; CRP, C reactive protein; PBMCs, peripheral blood mononuclear cell; VAT, visceral adipose tissue.

In order to find new associations between *LINE1* methylation and tumoral and inflammatory biomarkers, a Pearson correlation analysis was carried out between *LINE1* methylation and CEA, CA 19.9 (tumoral markers), insulin growth factor type 1 (IGF1), and C reactive protein (CRP) (inflammatory markers), according to the circulating levels of vitamin B12 (Figure 3). In the LVB_12_ group, *LINE1* methylation in tumor was inversely associated with CEA (r = −0.70; *p* = 0.003). In the HVB_12_ group, *LINE1* methylation in PBMCs and VAT were negatively associated with CRP (r = −0.34; *p* = 0.020 and r = −0.36; *p* = 0.014, respectively) and CEA was positively associated with CA 19.9 (r = 0.81; *p* < 0.001).

### 3.5. Regression Analyses between Methylation of LINE1 and Vitamin B12

To study the strength of the associations observed in the correlation analyses, we performed multivariable lineal regression analyses. We observed that *LINE1* methylation in the tumor area was positively and significantly associated with neoadjuvant therapy in both the LVB_12_ and HVB_12_ groups (Table 3). When we stratified by sex, and studied only men, only the LVB_12_ group showed a positive and significant association between *LINE1* methylation and neoadjuvant therapy (*p* = 0.006).

Finally, a multivariable logistic regression model was constructed to identify those factors that were independently associated with circulating vitamin B12 levels in CRC patients. In this model, after adjusting for age and sex, we found that lower *LINE1* methylation in the tumor area (odds ratio (OR) = 0.82, confidence interval (CI) 95% 0.70 to 0.95; *p* = 0.01 for both sexes; and OR = 0.74, CI 95% 0.57 to 0.90; *p* = 0.008 for only men), but not in PBMCs and VAT (data not shown), better explained the circulating levels of vitamin B12 in CRC patients, with a Nagelkerke r^2^ of 0.22 and 0.35 for only men (Table 4).

## 4. Discussion

In our study, we observed that serum vitamin B12 levels were associated with global DNA methylation in CRC patients, suggesting a potential role of vitamin B12 on the metabolism of DNA methylation in CRC. In line with previous findings about DNA methylation and vitamin B12 [5,12,18], we found that high circulating vitamin B12 levels were associated with decreased methylation of *LINE1* in tumor CRC. *LINE1* is typically found hypomethylated in CRC [19,20], and tumor hypomethylation of *LINE1* was also significantly associated with worse overall survival and poor prognosis compared to patients with a higher level of *LINE1* methylation in tumor [20]. On the other hand, a study found that patients with advanced CRC and decreased circulating vitamin B12 concentration had significantly better overall survival and progression time than patients with normal or higher circulating vitamin B12 [21]. This observation was further detected from a retrospective study conducted in patients with metastatic CRC [22]. Thus, our results showed that circulating vitamin B12 levels could have a role in DNA methylation in CRC subjects. Therefore, vitamin B12 levels could be used as a prognostic factor in CRC, as its association with *LINE1* methylation in the tumor area from CRC patients could place it as a promising factor in the prognosis of CRC.

Several epidemiological studies, meta-analyses, and supplemental studies have tried to determine the specific role of circulating, dietary, and/or supplemental vitamin B12 with CRC risk, although the results are still controversial. For example, two meta-analyses of prospective and case-control studies and a case-control study evaluated the association between circulating and vitamin B12 intake and CRC risk, which concluded that dietary vitamin B12 was inversely associated with the risk of CRC [23,24,25]. However, other studies did not find significant associations between CRC risk and neither dietary [26] nor serum vitamin B12 [27,28]. Another nested case-referent cohort study showed that high circulating levels of vitamin B12 were associated with lower risk of rectal cancer, whereas for colon cancer, this association seemed to be attenuated [8]. In contrast, a long-term follow-up study, in the framework of the B Vitamins for the Prevention of Osteoporotic Fractures (B-PROOF) Trial, concluded that vitamin B12 and folate supplementation were associated with an increased risk of CRC [11], which was further confirmed by another study [29]. Thus, data regarding the risk of CRC and vitamin B12 are not well understood, which might be due to the source of vitamin B12 from foods of animal origins potentially altering the risk of CRC [7]. In fact, consumption of white meat or poultry was found to be negatively associated with some types of cancers, whilst red meat consumption was significantly associated with increased risk of cancer [30]. Consequently, more epidemiological studies and clinical trials are needed to clarify the casual role of vitamin B12 in CRC, as this evidence should be considered in the management of the implication of vitamin B12 on CRC risk.

Our results also showed that *LINE1* methylation in VAT was associated with inflammatory markers and insulin metabolism in the HVB_12_ group. A study reported that vitamin B12 was related to the growth hormone (GH)/IGF1 axis, which is considered as an essential vitamin that regulates growth through this axis [31]. Indeed, there is promising evidence of the relationship between vitamin B12 and both insulin metabolism and resistance [18,32,33]. As far as we know, the insulin/IGF system is a major factor for the pathogenesis of CRC, as components of this system contribute to the transformation of normal colon epithelial cells [34]. Thus, a dysfunctional VAT could participate in the pathogenesis of CRC through the insulin pathway, in which vitamin B12 could play a relevant role in epigenetic regulation. Furthermore, a comprehensive understanding of the biological functions of vitamin B12 in VAT—through insulin metabolism and DNA methylation—could support the idea of the implication of vitamin B12 in CRC in larger studies, considering glucose, insulin, and IGF1 levels as confounders in the management of CRC risk and serum vitamin B12. Our study also suggested a link between DNA methylation and neoadjuvant therapy according to the serum vitamin B12. The significant correlation between *LINE1* and neoadjuvant therapy in only the LVB12 group suggests a potential role for vitamin B12 and DNA methylation as a biomarker of response [35].

There are several mechanisms by which vitamin B12 could be associated with CRC, although the most solid evidence is related to the role of vitamin B12 in one-carbon metabolism, which is required for DNA methylation [2]. Indeed, there are several studies that found an association between vitamin B12 and methylation of key genes in CRC, such as cyclin-dependent kinase inhibitor 2 A (p16), a tumor suppressor gene; and methylguanine methyltransferase (MGMT), a DNA-repairing protein or homeobox A 4 (*HOXA 4*)—its overexpression leads to CRC [12,36,37]. Although the evidence about the implication of vitamin B12 on DNA methylation and CRC is scarce, our results suggest that vitamin B12 could act as an epigenetic mediator that influences some pathways of cell cycles and proliferative properties of CRC, based on the epigenetic landscape.

This study has certain limitations that must be mentioned. The relatively small size of included patients in our study may mask other conflicting factors. However, the strengths of the study were the careful design and the inclusion of several biological and tissue samples for the study of methylation, which provide an extensive exploratory insight for the global DNA methylation in CRC and vitamin B12 levels.

## 5. Conclusions

In conclusion, the present study reported an inverse association between DNA methylation and vitamin B12 levels in CRC subjects. Increased vitamin B12 levels (i.e., higher than the 25th percentile) were associated with hypomethylation of *LINE1*, which suggests that vitamin B12 may have a role in epigenetics in CRC. Additional analyses also suggested that vitamin B12 could be associated with DNA methylation and insulin metabolism, suggesting a possible link for the insulin pathway in CRC. Hence, serum vitamin B12 could be used as a strong factor to consider in the management of CRC via DNA methylation.

## Figures and Tables

**Table 1:** Baseline general characteristics of CRC patients according to their 25th percentile of vitamin B12.

Variables	Low Serum Vitamin B12	High Serum Vitamin B12
*n* (%)	19 (23.8)	61 (76.2)
Age (years)	68.21 ± 10.33	67.38 ± 10.91
Sex (males/females)	16/3	21/40 *
BMI (kg/m^2^)	26.74 ± 3.02	27.53 ± 4.36
Fasting glucose (mg/dL)	134.63 ± 66.92	121.68 ± 52.07
Fasting insulin (μUI/mL)	4.95 ± 3.36	6.65 ± 5.36
HOMA-IR	1.62 ± 1.48	2.10 ± 2.05
Triglycerides (mg/dL)	167.95 ± 75.51	158.73 ± 82.13
Total cholesterol (mg/dL)	160.00 ± 24.30	175.47 ± 44.65
HDL-c (mg/dL)	37.36 ± 11.78	42.49 ± 14.64
LDL-c (mg/dL)	91.04 ± 25.12	106.28 ± 35.30 *
CEA (mg/dL)	5.88 ± 13.23	4.97 ± 8.17
CA 19.9 (U/mL)	18.63 ± 21.36	20.92 ± 30.96
IGF1 (ng/mL)	97.66 ± 39.92	137.56 ± 74.91 *
CRP (mg/L)	6.03 ± 4.56	9.77 ± 12.99
Vitamin B12 (pg/mL)	199.79 ± 49.14	507.02 ± 337.83 **

Low and high vitamin B12 groups are divided under the 25th percentile of vitamin B12. Data are expressed as mean ± standard deviations or *n* (percentage). Asterisk indicates significant difference between low and high vitamin B12 groups, according to Welch’s two sample tests (* *p* < 0.05; ** *p* < 0.001). Chi squared test was used for variables expressed as percentage (* *p* < 0.05). Abbreviations: CRC: Colorectal cancer; BMI: Body mass index; HOMA-IR: Homeostasis model assessment of insulin resistance; HDL-c: High-density lipoprotein cholesterol; LDL-c: Low-density lipoprotein cholesterol; CEA: carcinoembryonic antigen. CA 19.9: carbohydrate antigen 19.9; IGF1: insulin growth factor type 1; CRP: C reactive protein.

**Table 2:** Correlation between anthropometric and biochemical parameters and *LINE1* methylation according to serum vitamin B12 levels in CRC patients.

*LINE1* Methylation	Age	BMI	Glucose	Insulin	HOMA-IR	TG	TC	LDL-c	HDL-c
Low vitamin B12 group									
PBMCs	0.13	−0.07	−0.19	−0.41	**−0.53**	0.10	0.36	0.16	-0.26
VAT	0.49	−0.10	−0.20	0.31	0.22	−0.32	−0.49	−0.09	0.18
Tumor area	−0.03	−0.24	−0.01	−0.04	−0.06	−0.04	−0.27	0.26	−0.02
High vitamin B12 group									
PBMCs	−0.09	0.03	0.04	0.10	0.10	−0.12	−0.21	−0.16	0.16
VAT	<0.01	−0.27	−0.22	**−0.32**	**−0.31**	0.02	−0.02	−0.03	0.12
Tumor area	−0.02	0.15	−0.16	0.25	0.19	0.26	0.08	0.14	0.17

Low and high vitamin B12 groups are divided by their 25th percentile of vitamin B12. Data are expressed as correlation coefficient. Bold font indicates significant correlation according to the Pearson correlation test (*p* < 0.05). No correlation remained significant thereafter. Abbreviations: CRC, Colorectal cancer; PBMCs, Peripheral blood mononuclear cells; VAT, Visceral adipose tissue; *LINE1*, Long interspersed nuclear element-1; BMI, Body mass index; HOMA-IR, Homeostasis model assessment of insulin resistance; HDL-c, High density lipoprotein-cholesterol; LDL-c, low density lipoprotein-cholesterol; TC, total cholesterol; TG, triglycerides.

**Table 3:** Multivariable lineal regression analysis with *LINE1* methylation as dependent variables in CRC patients.

Variables	Both Sexes		Men Only	
	Low Vitamin B12 β (SE)	High Vitamin B12 β (SE)	Low Vitamin B12 β (SE)	High Vitamin B12 β (SE)
*LINE1* methylation: PBMCs	r^2^ = 0.32; *p* = 0.07	r^2^ = −0.10; *p* = 0.99	r^2^ = 0.38; *p* = 0.17	r^2^ = −0.30; *p* = 0.51
Age	0.09 (0.04)	−0.00 (0.03)	0.22 (0.04)	−0.08 (0.05)
Sex	−3.48 (1.16) *	−0.11 (0.71)	NA	NA
BMI	0.05 (0.15)	0.00 (0.08)	0.00 (0.13)	0.11 (0.18)
Neoadjuvant therapy	−0.07 (1.01)	−0.16 (0.78)	0.13 (0.86)	−0.15 (1.67)
*LINE1* methylation: tumor area	r^2^ = 0.51; *p* = 0.03 *	r^2^ = 0.18; *p* = 0.01 *	r^2^ = 0.64; *p* = 0.03 *	r^2^ = 0.32; *p* = 0.46
Age	0.05 (0.10)	0.03 (0.07)	0.07 (0.12)	0.12 (0.16)
Sex	2.63 (2.80)	−0.57 (1.65)	NA	NA
BMI	−0.85 (0.39)	0.16 (0.18)	−0.88 (0.43)	−1.53 (1.61)
Neoadjuvant therapy	7.80 (1.99) *	5.99 (1.87) *	7.87 (2.12) *	8.92 (5.91)

Lineal regression analysis with *LINE1* methylation in PBMCs, VAT and tumor area as dependent variable and neoadjuvant therapy as independent variables and corrected for age, sex and BMI. Low and high vitamin B12 groups are divided under the 25th percentile of vitamin B12. Data are expressed as β (SE)of methylation (%). Asterisk indicates significant difference between low and high vitamin B12 groups, according to the linear regression analysis (* *p* < 0.05). Abbreviations: CRC, Colorectal cancer; PBMCs, peripheral blood mononuclear cell; *LINE1*, Long interspersed nuclear element-1; SE, Standard error; BMI, body mass index; r^2^: coefficient of determination; NA, not applicable.

**Table 4:** Factors associated with vitamin B12 variation in CRC patients.

Variables	Both Sexes	Men Only
	OR (CI 95%) r^2^ = 0.22	OR (CI 95%) r^2^ = 0.35
Age	1.01 (0.94–1.07)	0.98 (0.91–1.06)
Sex	2.15 (0.51–11.91)	NA
Tumor *LINE1* methylation	0.82 * (0.70–0.95) *	0.74 (0.57–0.90) *
CEA	0.96 (0.91–1.03)	0.90 (0.77–1.00)

Vitamin B12 was used as dependent variable (the value below the 25th percentile of vitamin B12 (0) vs. value above the 25th percentile of vitamin B12 (1)). Data are expressed as OR (CI 95%). Asterisk (*) indicates significant value according to the logistic regression analysis. Abbreviations: CRC, Colorectal cancer; OR, odds ratio; CI, confidence interval; CEA, carcinoembryonic antigen; *LINE1*, Long interspersed nuclear element-1; NA, not applicable.

## Supplementary Materials

The following are available online at https://www.mdpi.com/2072-6643/12/11/3567/s1, Table S1: Pathological characteristics of CRC patients according to their 25th percentile of vitamin B12; Table S2: Data of CpG analyzed and primer sequences of selected promoters.
